# In Silico Finite Element Modeling of Stress Distribution in Osteosynthesis after Pertrochanteric Fractures

**DOI:** 10.3390/jcm11071885

**Published:** 2022-03-28

**Authors:** Jacek Lorkowski, Mieczyslaw Pokorski

**Affiliations:** 1 Department of Orthopedics, Traumatology, and Sports Medicine, Central Clinical Hospital of the Ministry of the Internal Affairs and Administration, 137 Woloska Street, 02-507 Warsaw, Poland; jacek.lorkowski@gmail.com; 2 Institute of Health Sciences, Opole University, 68 Katowicka Street, 45-060 Opole, Poland

**Keywords:** dynamic hip screw, finite element modeling, gamma nail, pain perception, pertrochanteric fracture, osteosynthesis, rehabilitation

## Abstract

A stabilization method of pertrochanteric femur fractures is a contentious issue. Here, we assess the feasibility of rapid in silico 2D finite element modeling (FEM) to predict the distribution of stresses arising during the two most often used stabilization methods: gamma nail fixation (GNF) and dynamic hip screw (DHS). The modeling was based on standard pre-surgery radiographs of hip joints of 15 patients with pertrochanteric fractures of type A1, A2, and A3 according to the AO/OTA classification. The FEM showed that the stresses were similar for both GNF and DHS, with the medians ranging between 53–60 MPa and consistently lower for A1 than A3 fractures. Stresses also appeared in the fixation materials being about two-fold higher for GNF. Given similar bone stresses caused by both GNF and DHS but shorter surgery time, less extensive dissection, and faster patient mobilization, we submit that the GNF stabilization appears to be the most optimal system for pertrochanteric fractures. In silico FEM appears a viable perioperative method that helps predict the distribution of compressive stresses after osteosynthesis of pertrochanteric fractures. The promptness of modeling fits well into the rigid time framework of hip fracture surgery and may help optimize the fixation procedure for the best outcome. The study extends the use of FEM in complex orthopedic management. However, further datasets are required to firmly position the FEM in the treatment of pertrochanteric fractures.

## 1. Introduction

Hip fractures are common in the elderly. The number of cases increases every year due to the lengthening period of physical activity and lifespan [1,2]. Pertrochanteric fractures represent those that extend from the base of the femoral neck to a point located below the lesser trochanter. Such fractures also occur in young people due to exposure of the bone to a strong force [3]. In the elderly, a low-energy trauma, e.g., a simple fall, may result in an osteoporotic fracture. Additionally, cardiovascular, metabolic, and neurological comorbidities increase the propensity for femur fractures [4]. Fractures in the elderly are a source of protracted pains, difficult to treat, may reoccur, and involve a greater mortality rate. The treatment of pertrochanteric fractures is mostly performed surgically using various types of standard stabilization methods. It is believed that the time between the injury and surgical treatment is an essential factor for the outcome. The recommendation is to perform surgery up to 48 h after the injury, which reduces the risks of complications and mortality [5,6].

Currently, the gamma nail system (GNS) and the dynamic hip screw (DHS) are the most widespread stabilization methods used [7,8]. The criteria of choosing one or the other are not fully clear. So far, the decision-making process depends on the diagnostic imaging (fracture pattern and underlying bone stock) supplemented by the surgeon’s empirical experience. Unfortunately, the selection of the treatment method is burdened with uncertainties and, consequently, errors, leading to serious adverse events. The most notable adverse effect is an implant failure due to various reasons with a rate of occurrence of 0.5% in A1, 1.3% in A2, and 9.7% in A3 types of fractures (AO/OTA classification) [9]. Artificial intelligence (AI) that rapidly develops in the field of medicine holds promise to optimize the decision-making process in the perioperative stage. One such method using AI algorithms is finite elements modeling (FEM). It allows determining the distribution of compression stresses occurring within the bone and at the bone-fixation material interface. The method is based on in silico processing of X-ray, CT, or MRI scans [10,11]. The advantage of the FEM lies in the pre-operative prediction of the exact place and size of stresses in contrast to the hitherto used assessment occurring after surgical treatment of a fracture [12,13]. Therefore, in this study, we performed in silico virtual FEM using pre-surgery radiographs of pertrochanteric fractures to predicate stresses arising after stabilization surgery using GNF and DHS systems. The rationale was that FEM pursued before surgery could help predict which implant behaved better given the stresses produced and thus minimize a chance of post-operative complications by avoiding the use of the other.

## 2. Materials and Methods

### 2.1. Patients

This is a retrospective study based on pre-operative X-rays of 15 patients aged 82 ± 10 (SD) years, ranging 65–98 years (nine women and six men), randomly selected from 119 cases of pertrochanteric fractures surgically treated by one orthopedic surgeon between 2015–2019. The right trochanter region of the femur was injured in eight and the left in seven patients. The X-rays and patient data were received from anonymized medical files. There were five patients with type A1, A2, and A3 pertrochanteric fractures each, according to the AO/OTA classification [14]. Type A1 and A2 patients were stabilized with GNF and DHS, two and three cases in either type, and the five A3 patients were only stabilized with GNF according to the current clinical consensus. The fracture stabilization procedures were performed under spinal subarachnoid anesthesia. Analgesics were administered to patients as required in the perioperative period. Results of all surgeries were good/fair for patients aged below/over 80 years according to the Harris Hip Score (HHS) measure of dysfunction [15], persisting so at a one-year follow-up. Characteristics of patients subjected to GNF and DHS stabilizations are shown in Table 1. Patients stabilized with GNF tended to be older than those with DHS; medians of 88 and 80.5 years of age, respectively. The median duration of hospitalization was comparable amounting to 7 days. However, the surgery time was appreciably shorter for the GNF patients with the median of 30 vs. 45 min for DHS, *p* ≤ 0.05. From a technical standpoint, the tissue dissection also was shorter for GNF amounting to 8–10 cm vs. 10–12 cm for DHS. Additionally, patients stabilized with GNF were instructed to start partial loading of the operated limb after two weeks and full loading, unprotected with elbow crutches, after about four months as opposed to six weeks and five months, respectively, after DHS. An exception concerned A3 patients stabilized with GNF who were allowed the unprotected loading after five months on par with the DHS-stabilized patients. The post-treatment results of HHS were comparable, with the medians of 77–79 points in both stabilization methods. The HHS is a measure of dysfunction, with a higher score indicating a better outcome for the patient. A major domain of the scale concerns pain perception, with the score ≥ 44 indicating an ignorable level of pain. Fracture stabilization had no appreciable effect on pain. In most patients, the pain was mild, with a median of 30 points, little affecting usual daily activities, and requiring an occasional intake of aspirin or paracetamol pills at most.

### 2.2. Finite Element Modeling (FEM)

The FEM was based on a rapid 2D in silico analysis performed ex post facto as an imaginary surgery planning without any involvement of patients. The analysis used the original X-rays taken in the anteroposterior position of the pelvis and the proximal end of the femur with a visible transtrochanteric fracture during hospital admission of patients. Representative examples of such radiographs are shown in the Supplementary Materials of this article. Distributions of stresses arising in the proximal end of the femur and stabilization systems were assessed. The analysis time for one patient was about 45 min.

The method consisted of assigning 256 material attributes (shades of the grayscale bitmap of X-ray images) to the FEM model using the CT2FEM v1.0 software (M. Mrzyglod, Cracow University of Technology, Kraków, Poland). A 2D section area of the bone was modeled using eight-node solid linear finite elements of 1 mm size grouped by material properties. The FEM was based on radiological images and the mesh was not optimized, given the assumption of rapid modeling for the real-time simulation in the perioperative situation. The only possible optimization, in this case, was the use of high-order elements (p-method). The boundary conditions included the fixation at the model’s base and the load in line with the gravitational force at its top. The model was performed in the fixed frontal plane congruent with the 2D assumptions and thus without the evaluation of torsion.

The model included the areas made up of healthy bone tissue, the articular cartilage of the hip joint, soft tissues inside the medullary cavity, and the fixation material. A simplified, homogeneous description of the tissue properties was adopted for numerical tests. For the compact bone, Young’s modulus was E = 5 GPa and Poisson’s number ν = 0.32, and for the spongy bone, E = 3 GPa and ν = 0.32. Correspondingly, for the articular cartilage, E = 600 MPa and ν = 0.42, for soft tissues, E = 0.6 MPa and ν = 0.42, and for the joining material, E = 210 GPa and ν = 0.30 were assumed. For each model, static analysis was performed for the adopted load pattern (weight load was distributed evenly over the cross-section of the pelvic bone). The processing was supported by taking away all degrees of freedom in the nodes at the bottom edge of the model. The average patient’s weight was assumed as 70 kg. Therefore, the FEM was loaded with a force corresponding to an average load of 1 N/1 mm^2^, the vector of which was consistent with the limb axis [10]. The spatial distribution of stresses was adopted as a measure of the bone or fixation material effort, rather than a single tension component, before fracturing or yielding according to the Huber–Mises–Hencky hypothesis [16,17].

Stresses were expressed in MPa and shown as medians and minimum-maximum values for the three types of fractures according to the AO/OTA classification. The distribution of some group data, based on the Shapiro–Wilk test, was not normal. Comparisons of stress differences among the three types of fractures stabilized using either system or between the corresponding types of fractures stabilized using the same system were based on the Kruskal–Wallis test with the Bonferroni correction for pairwise comparisons. Outcomes of the GNF and DHS stabilization procedures were compared using the Mann–Whitney U two-sample rank-sum test for independent samples. The null assumption was that the medians of the groups were equal. A *p*-value ≤ 0.05 two-tailed defined statistically significant differences. Statistical analysis was performed using a commercial SPSS v19.0 statistical package for Windows (SPSS Inc.; Chicago, IL, USA).

## 3. Results

### 3.1. Distribution of Stresses in Bone Tissue

**Figure 1:**
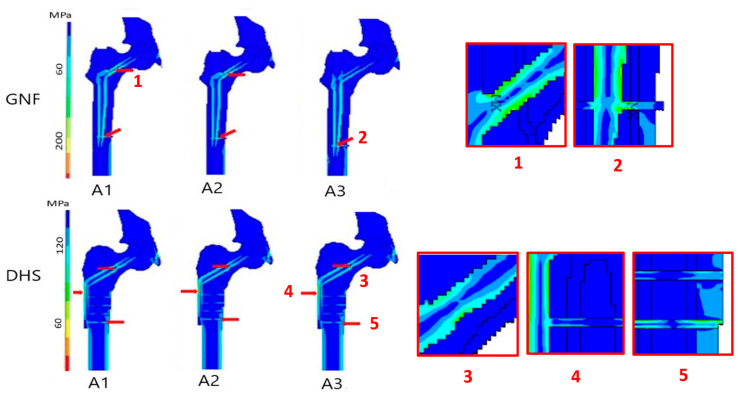
Images presenting the simulation of stress distributions in the bone tissue obtained by rapid finite element modeling (FEM) after pertrochanteric fracture osteosynthesis stabilized with gamma nail fixation (GNF) and dynamic hip screw (DHS) systems. The A1, A2, and A3 fracture types are presented (AO/OTA classification). The squares on the righthand side represent the enlarged visualization of the most relevant stresses depicted by the corresponding numbers in either stabilization system. The unnumbered red arrows in the lefthand side of the figure indicate that the stress distribution was grossly akin to the numbered counterparts.

Figure 1 shows representative examples of the FEM simulation of stresses arising in the bone tissue of the femoral shaft. Individual types of pertrochanteric fractures are presented according to the AO/OTA classification using the GNF and DHS stabilization systems. The highest stress was observed in the medial layer of the bone cortex. High stresses also were present about the lower screws of the DHS stabilization.

The median bone stresses were 54.0 MPa to 58.6 MPa for GNF, and 52.8 MPa to 60.0 MPa for DHS, with lower values for A1 and higher values for A3 fractures. These differences between the fracture types were significant with large effect sizes in either stabilization system, rejecting the null assumption of equal medians. However, the differences between the corresponding types of fracture between the GNF and DHS systems were insignificant. The *Z*-score, comparing the rank mean of a group to the overall rank mean, was close to zero, which shows that each of these type groups had an equal probability of containing the highest value (Table 2).

Bone stresses in the corresponding fracture types were 2–3-fold lower than those arising in the fixation materials in both GNF and DHS stabilization procedures.

### 3.2. Distribution of Stresses in Fixation Material

The medians of stresses in fixation materials used for the GNF and DHS stabilization systems for the three pertrochanteric fracture types are displayed in Table 3. Stresses were significantly higher in A3 fractures. For GNF, stresses were increased in the central part of the neck screw, the distal end of the nail, and the locking screw. For DHS, they were increased in the plate area, the middle part of the neck screw, and the lowest of cortical screws. Notably, tensions were nearly twice higher in GNF when compared to the DHS stabilization system in all fracture types (*p* = 0.012).

## 4. Discussion

This study aims to simulate the distribution of stresses arising in and around the site of osteosynthesis after surgical management of pertrochanteric fractures. The issue is essential for choosing the optimal fixation system during surgery to get the best outcome minimalizing the rate of failed bone union, dislocations, healing in malposition with possible pseudoarthrosis, refractures, and reoperations [5]. The fixation provides a ‘biomechanical silence’ at the fracture site as these are anchored methods of ostheosynthesis, and the movement rather than stress is responsible for the pain. The stabilization systems mostly used, GNF and DHS, essentially have similar rates of positive outcomes. DHS is an extramedullary fixation with a single cephalic screw [18,19] while GNF is an intramedullary fixation [20,21]. The factors influencing the choice of a stabilization system are not always fully clear and sometimes steered by an orthopedist’s experience or system’s availability, which escapes precise aims. Therefore, in this study, we set out to counter compare stresses arising in the bone tissue and fixation material after osteosynthesis of pertrochanteric fractures managed with GNF and DHS simulated by in silico modeling. Fractures were stratified into A1, A2, and A3 types to the AO/OTA classification [14]. The findings were that bone stresses were similar in the corresponding types of fractures in both systems. Generally, the stresses arose in cortical layers of the medial side of the femur and below the lower screws followed by lateral stress in the cortical layer of the femoral shaft below the implant, and to a lesser extent in the trochanteric region and the femoral neck and head. There were, however, consistent differences among the three types of fractures in either stabilization system, with the lowest stresses in A1 and highest in A3 types, which speaks for a lower probability of the shaft fracture under the implant in A1 type. In this context, it is worth noting that type A1 is a stable oblique trochanteric fracture at the base of the femoral neck. The fracture is at a variable angle of the bend of the sagittal plane and the horizontal plane. By contrast, type A2 and A3 fractures are in the intermediate sagittal/horizontal planes, are often unstable or multi-fragmented, and type A3 may also be wedge fractures or assume intertrochanteric reverse oblique shape. The enhanced complexity of A2 and A3 fractures may translate into greater pertrochanteric stresses after osteosynthesis, which at the same time validates the viability of in silico FEM simulation of the stress distribution presented in this study.

Stresses arising in stabilizing materials were nearly two-fold greater than bone stresses across A1-A3 fractures in both GNF and DHS systems. The stresses were in the middle part of the neck screw and the lower part of the nail and locking screw in GHF, and the central part of the neck screw, the plate, and lower cortical screws in DHS. Lower stresses occurred in the central part of the neck screw in A1 and A2 fractures in both systems concentrating about the neck screw, as opposed to the distal part of the nail in A3 fractures.

The present findings are generally in line with those of other studies performing in silico modeling and clinical observations of orthopedic surgeons. Jiang et al.’s (2017) [22] modeling has shown the presence of higher stresses at the nail border and the neck screw using different types of nails, which was confirmed during real-life orthopedic surgery by [23]. Higher stresses have been reported about the locking screw and the lower part of the nail, being greater in GNF than DHS [24,25]. As with the present findings, modest bone stress has been described at the implant level compared to the implant itself. Such results are consistent in both 2D and more elaborate 3D models [26]. The 2D FEM appears well suited and superior for perioperative modeling. The method is prompt. In the present study, it took 30–45 min from the input of data to the display of the stress distribution.

Based on the present findings and our extensive orthopedic experience, we submit that GNF has an edge over DHS due to intracortical placement in the bone helping stabilize a difficult osteosynthesis in brittle bone and multi-fragmented fractures, lower mass of fixation material and its simpler structure, a smaller incision and extent of surgical approach, and shorter surgery time. These features, enumerated in Table 1, are liable to prevail over smaller stresses arising in more massive and extensive DHS fixation material. The extent of soft tissue damage was less in GNF when compared to DHS. Patients stabilized with GNF started to fully load the limb operated on a month earlier than those with DHS despite being, on average, about four years senior. Thus, the overall surgery trauma was less in GNF. This aligns with the observations of other authors [27]. Studies also indicate a generally lower intraoperative blood loss using GNF [28,29,30]. The choice between GNF and DHS stabilizations of femur fractures can hardly be influenced by the post-treatment motion-related pain perceived by patients as it is rather mild, irrespective of the system. Few differences in post-operative analgesic requirements also were noticed in other studies [31]. Thus, this choice remains difficult and contains an indispensable empiric element of the surgeon’s experience and individual circumstances related to the patient’s status, fracture particularities, and operative conditions. FEM results, albeit supplementary, may facilitate the decision-making in the implementation of orthopedic management.

Physicians have been using fixation to treat fractures from Hippocrates’ time. The stabilization design and biomechanics have changed over the years, but the principles remain the same. The primary goal of bone stabilization is to maintain the length, alignment, and rotation of the fracture. With absolute fracture stability, the bone will undergo primary intramembranous bone healing. Relative fracture stability, on the other hand, results in secondary endochondral bone healing [32]. These rules also apply to pertrochanteric fractures. The present study demonstrates that in silico 2D FEM is a viable loop of research-based simulation that helps predict the magnitude of stress possibly arising in osteosynthesis of pertrochanteric fractures. The method refers to the current surge in the application of AI algorithms for personalized medicine based on individual patient characteristics [33,34,35,36].

In silico modeling uses AI algorithms that allow a program to be “thought” by raw data [37]. Olczak et al. [38] have tested the possibility of using “deep learning” to analyze X-ray images to detect various skeletal elements in limb fractures. The authors find that AI-based algorithms are as effective as the radiologist’s eye and any errors are mostly caused by the poor image quality. Similar schemes have also been tested in the diagnostics of breast cancer or SARS-CoV-2 infections using magnetic resonance or computer tomography images [39,40]. In orthopedics, classic X-ray remains the diagnostic basis due to the prompt execution, the ease of interpretation due to a relatively limited amount of data, and a low-cost performance as in the 2D FEM. Results are interpreted by the investigator performing the modeling. The presumption is that AI-supported “deep learning” will enable automated surgeon information about the best treatment method soon.

In this study, we did not use 3D FEM that is considered to reflect best the bone and musculoskeletal systems [17,18]. Such a procedure, however, often takes days of a multi-person workforce and appears suitable for scientific research or novel implant designs rather than clinical practice. Our goal was to present the feasibility of a simple fast-performing tool conducive to rational decision-making by a surgeon in the perioperative environment of the management of pertrochanteric fractures. To this end, we believe we have demonstrated that, despite inherent limitations due to computational simplifications not always reflecting real-world conditions, the in silico 2D FEM based on readily available X-rays could be helpful, particularly when CT images are often unavailable in the ‘emergency’ mode [41,42]. Additionally, the time elapsing from the injury to surgery is not indifferent in proximal femur fractures as it increases secondary fractures, mortality, and socioeconomic aspects, particularly in the elderly who suffer the fractures most often [5].

Recently, a modified, technically demanding stabilization system, proximal femoral nail with anti-rotation (PFNA), has been introduced in the orthopedic management of femur fractures. The PFNA appears superior in some perioperative respects, such as lower blood loss, but inferior in maintaining the abductor muscle strength when compared with DHS and GNF [43]. Here, we did not model the stress distribution arising in response to PFNA, which may be considered a weakness of the study. However, PFNA, akin to other modifications performed, has not displaced the time-proven DHS and GNF given the comparable functional outcomes of all these methods of pertrochanteric fractures stabilization [7,8,27,44]. There are other limitations of this study. Namely, the number of cases investigated with the three fracture types was small, and larger samples would be needed to obtain robust results. We also failed to consider the femoral bone stock which is an important factor in shaping the strength available to support the implant; such data cannot be deduced from standard X-ray images. Finally, we used images in the anterior-posterior projection, skipping the radiographs taken in the sagittal or axial plane. Despite these limitations, we believe that we have shown the feasibility and effectiveness of FEM which potential usefulness in perioperative fracture management should be confirmed in broader prospective investigations.

## 5. Conclusions

In silico 2D FEM is a viable and effective perioperative method that helps predict the distribution of compressive stresses after osteosynthesis of pertrochanteric fractures. Bone stresses appear similar using gamma nail and dynamic hip screw stabilization systems, but stresses arising in the fixation material are higher in the latter system. Nonetheless, we opine that the gamma nail stabilization may hold an edge due to less demanding and extensive surgery and faster patient mobilization afterward. The relative promptness and ease of in silico 2D modeling fit well into the rigid operative time framework of trochanteric surgery. The modeling may facilitate the decision of selecting the optimal surgical technique for load transmission across the femur but cannot substitute the empiric element of the surgeon’s experience and consideration of individual patient medical circumstances.

## Figures and Tables

**Table 1:** Characteristics of patients stabilized with gamma nail fixation (GNF) and dynamic hip screw (DHS) surgeries after pertrochanteric fractures.

Stabilization	Pertrochanteric Fracture Types—AO/OTA Classification				
	Age (Years)	HHS (Score)		Surgery Time (min)	Hospitalization (Days)
		Total	Pain		
GNF (*n* = 9)	88 (69–98)	78.8 (61.8–95.9)	30 (20–40)	30 (20–50)	7 (4–12)
DHS (*n* = 6)	80.5 (65–91)	76.7 (60.8–95.9)	30 (20–40)	45 (30–50)	7 (4–18)
Z-score	1.24	0.00	0.06	−1.94 *	0.12
U (critical value ≤10)	16	27	27	10 *	26

Data are medians (min-max); HHS, Harris Hip Score. Mann-Whitney rank-sum test: U-value, difference between the two rank totals (the smaller the U the less likely it is that a difference occurred by chance); Z-score, comparison of the rank means in a group to the overall rank mean (closeness to 0 indicating an equal probability of containing the highest value). * Significant results at *p* ≤ 0.05 two-tailed.

**Table 2:** Simulation of stress distribution in the bone tissue after pertrochanteric fracture osteosynthesis stabilized with gamma nail fixation (GNF) and dynamic hip screw (DHS) systems.

Stabilization Method	Pertrochanteric Fracture Types—AO/OTA Classification			*p* ^1^	*η* ^2^	Significant Difference of Mean Ranks of Pairs *
	A1	A2	A3			
GNF (MPa)	54.0 (48.4–56.5)	55.6 (52.4–58.5)	58.6 (56.4–59.5)	0.024	0.46	A1–A3
DHS (MPa)	52.9 (49.3–55.5)	54.9 (51.4–57.2)	60.0 (56.7–64.6)	0.008	0.64	A1–A3, A2–A3
*p* ^2^	0.401	0.674	0.834			
Z-score	0.84	0.42	−0.21			
U (critical value ≤ 2)	8	10	11			

Data are medians (min–max). *p* ^1^, *p*-value for the Kruskal–Walllis test; *η* ^2^, effect size (critical value is 0.30); * Bonferroni adjustment. *p* ^2^, *p*-value for the Mann–Whitney rank-sum test; U-value, difference between the two rank totals (the critical value at *p* < 0.05 was 2); Z-score, comparison of the rank means in a group to the overall rank mean (closeness to 0 indicating an equal probability of containing the highest value); results were not significant at *p* ≤ 0.05 two-tailed.

**Table 3:** Simulation of stress distributions in the fixation material after pertrochanteric fracture osteosynthesis stabilized with gamma nail fixation (GNF) and dynamic hip-screw (DHS) systems.

Stabilization	Pertrochanteric Fracture Types—AO/OTA Classification			*p* ^1^	*η* ^2^	Significant Differences of Mean Ranks of Pairs *
	A1	A2	A3			
GNF (MPa)	181.7 (157.3–195.5)	185.2 (173.5–196.4)	203.6 (195.4–213.7)	0.025	0.45	A1–A3
DHS (MPa)	91.5 (86.5–100.1)	111.2 (107.3–117.6)	124.9 (118.6–133.5)	0.002	0.88	A1–A2, A1–A3, A2–A3
U (critical value ≤ 2)	0	0	0			
Z-score	2.51	2.51	2.57			
*p* ^2^	0.012	0.012	0.012			

Data are medians (min-max); *p* ^1^, *p*-value for the Kruskal–Walllis test; *η* ^2^, effect size (the critical value is 0.30); * Bonferroni adjustment. *p* ^2^, *p*-value for *Z*-score in the Mann–Whitney rank-sum test; U-value, difference between the two rank totals (the smaller the U the less likely that a difference occurred by chance); Z-score, comparison of the rank means in a group to the overall rank mean (the further from 0 the smaller chance of an equal probability of containing the highest value); results were significant at *p* ≤ 0.05 two-tailed.

## Data Availability

The data presented in this study are available on request from the corresponding author.

## Supplementary Materials

The following supporting information can be downloaded at: https://www.mdpi.com/article/10.3390/jcm11071885/s1, Figure S1. A 79-year-old female who sustained a left pertrochanteric fracture (type A2—AO/OTA classification). A—fracture before surgery; B—one day after the stabilization with dynamic hip screw (DHS) system; and C—one-year follow-up. Figure S2. A 88-year-old female who sustained a right pertrochanteric fracture (type A3—AO/OTA classification). A—fracture before surgery; B—two days after the stabilization with gamma nail fixation (GNF) system; and C—one-year follow-up.
