# Supplementary material for: In Silico Finite Element Modeling of Stress Distribution in Osteosynthesis after Pertrochanteric Fractures
Source: J Clin Med. 2022 Mar 28;11(7):1885. doi: 10.3390/jcm11071885 (PMC8999495; doi:10.3390/jcm11071885)
Supplement: Supplementary file 1 [file jcm-11-01885-s001.zip › jcm-1627768-supplementary.pdf]

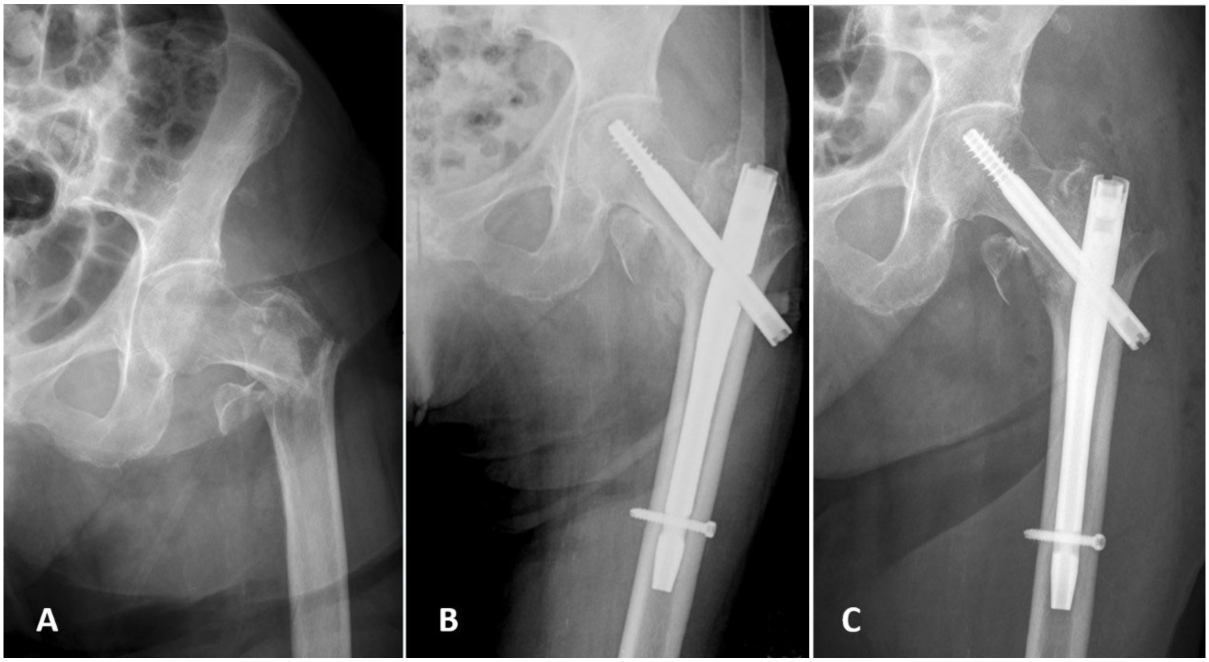

**Figure S1.** A 79-year-old female who sustained a left pertrochanteric fracture (type A2 - AO/OTA classification). **A**—fracture before surgery; **B**—one day after the stabilization with dynamic hip screw (DHS) system; and **C**—one-year follow-up.

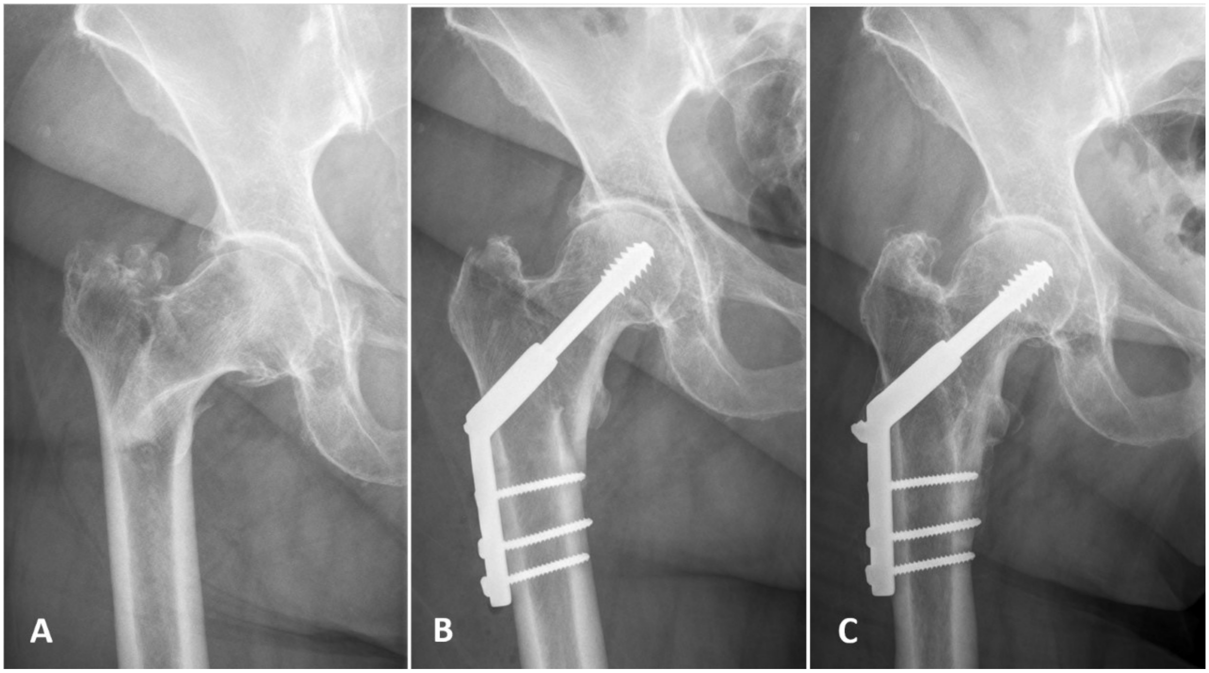

**Figure S2.** A 88-year-old female who sustained a right pertrochanteric fracture (type A3 - AO/OTA classification). **A**—fracture before surgery; **B**—two days after the stabilization with gamma nail fixation (GNF) system; and **C**—one-year follow-up.
